# Supplementary material for: Prediction of response to anti-cancer drugs becomes robust via network integration of molecular data
Source: Sci Rep. 2019 Feb 20;9:2379. doi: 10.1038/s41598-019-39019-2 (PMC6382934; doi:10.1038/s41598-019-39019-2)

# Supplementary File

**Prediction of response to anti-cancer drugs becomes robust via network integration of molecular data**

Marcela Franco^1^, [Marcela.Franco@ki.se](mailto:Marcela.Franco@ki.se);

Ashwini Jeggari^2^, [ashwini.jeggari@scilifelab.se](mailto:ashwini.jeggari@scilifelab.se);

Sylvain Peuget^1^, [sylvain.peuget@ki.se](mailto:sylvain.peuget@ki.se);

Franziska Böttger^1,3^, [f.bottger@vumc.nl](mailto:f.bottger@vumc.nl);

Galina Selivanova^1^ , [Galina.Selivanova@ki.se](mailto:Galina.Selivanova@ki.se), and

Andrey Alexeyenko^1,4*^, [andrej.alekseenko@scilifelab.se](mailto:andrej.alekseenko@scilifelab.se)

^1^ Department of Microbiology, Tumor and Cell Biology (MTC), Karolinska Institutet, Stockholm, Sweden.

^2^ Department of Cell and Molecular Biology, Karolinska Institutet, 171 77 Stockholm, Sweden.

^3^ Present Address: OncoProteomics Laboratory, Department of Medical Oncology, VU University Medical Center, 1081HV Amsterdam, The Netherlands

^4^ National Bioinformatics Infrastructure Sweden, Science for Life Laboratory, Box 1031, 17121, Solna, Sweden

* To whom correspondence should be addressed. Tel: +46 8 52481513; email: [andrej.alekseenko@scilifelab.se](mailto:andrej.alekseenko@scilifelab.se); address: SciLifeLab, Box 1031, 171 21 Solna, Sweden

## Supplementary tables

Supplementary Table 1. Comparative analysis of NEA sensitivity by using different AGS classes.

Similarly to Fig. 3 in the main text, fractions of significant Spearman correlations between features and drug sensitivity were evaluated. The distributions of fractions’ q-values (p-values adjusted for multiple testing) below 0.1 (FDR<0.1 by Benjamini and Hochberg, 1995) were compared in each pair of the five AGS categories: "significant", "significant.filtered.maxi", “significant.filtered.mini", "top.200", and "top.400". The tables display p-values of the Kolmogorov-Smirnov test. When the value is shown above the diagonal, then the category in the column header was significantly (after Bonferroni adjustment) better than the row category (i.e. contained more high fractions). Presence of p-value below the diagonal indicates that the row category was superior. When the difference was insignificant, the “NS” label is present both above and below the diagonal.

| **Cancer cell lines, gene copy number** | **significant** | **significant.filtered.maxi** | **significant.filtered.mini** | **top.200** | **top.400** |
| --- | --- | --- | --- | --- | --- |
| **significant** |  | NS | NS | 9.00E-06 | 1.40E-20 |
| **significant.filtered.maxi** | 1.10E-05 |  | 1.20E-07 | 3.30E-22 | 4.60E-54 |
| **significant.filtered.mini** | NS | NS |  | 0.00031 | 7.60E-17 |
| **top.200** | NS | NS | NS |  | 2.10E-05 |
| **top.400** | NS | NS | NS | NS |  |

| **Cancer cell lines, gene expression** | **significant** | **significant.filtered.maxi** | **significant.filtered.mini** | **top.200** | **top.400** |
| --- | --- | --- | --- | --- | --- |
| **significant** |  | NS | NS | 5.50E-05 | 1.30E-07 |
| **significant.filtered.maxi** | 6.40E-05 |  | NS | 2.40E-15 | 7.20E-20 |
| **significant.filtered.mini** | 5.20E-23 | 4.10E-09 |  | 2.60E-43 | 6.70E-51 |
| **top.200** | NS | NS | NS |  | NS |
| **top.400** | NS | NS | NS | NS |  |

| **TCGA, gene copy number** | **significant** | **significant.filtered.maxi** | **significant.filtered.mini** | **top.200** | **top.400** |
| --- | --- | --- | --- | --- | --- |
| **significant** |  | NS | NS | NS | NS |
| **significant.filtered.maxi** | 0.0041 |  | NS | NS | NS |
| **significant.filtered.mini** | NS | NS |  | NS | NS |
| **top.200** | 5.30E-05 | NS | NS |  | NS |
| **top.400** | 2.30E-09 | NS | NS | NS |  |

| **TCGA, gene expression** | **significant** | **significant.filtered.maxi** | **significant.filtered.mini** | **top.200** | **top.400** |
| --- | --- | --- | --- | --- | --- |
| **significant** |  | 0.00059 | NS | NS | NS |
| **significant.filtered.maxi** | NS |  | NS | NS | NS |
| **significant.filtered.mini** | NS | NS |  | NS | NS |
| **top.200** | 1.00E-04 | 4.60E-15 | 2.40E-09 |  | NS |
| **top.400** | 3.90E-17 | 1.20E-39 | 5.50E-27 | 3.00E-04 |  |

Supplementary Table 2. Feature classes that consistently revealed features associated with anti-cancer drug sensitivity (selected individual features are presented in the main text).

| Screen | Drug | Feature class in drug screen | Set | Type | Feature class in TCGA | Survival** | Follow-up time, days | Rank correlation between  i*n vitro* and TCGA | P-value of rank correlation   1. rank correlation |
| --- | --- | --- | --- | --- | --- | --- | --- | --- | --- |
| CTD | gemcitabine | top.400.affymetrix_cgp | BLCA | PWNEA | top.400.illuminahiseq_rnaseqv2 | RFS | 2141 | 0.262 | 1.2e-06 |
| CTD | gemcitabine | top.400.affymetrix_cgp | BLCA | PWNEA | top.400.illuminahiseq_rnaseqv2 | RFS | 4283 | 0.262 | 1.2e-06 |
| CTD | dexamethasone | top.400.affymetrix_ccle | GBM | PWNEA | top.400.agilent | OS | 1940 | 0.365 | 4.5e-12 |
| CTD | dexamethasone | top.400.affymetrix_ccle | GBM | PWNEA | top.400.agilent | OS | 3881 | 0.379 | 5.5e-13 |
| CTD | dexamethasone | top.400.affymetrix_ccle | GBM | PWNEA | top.400.agilent | OS | 776 | 0.264 | 1.0e-06 |
| CCLE | topotecan | top.200.affymetrix_ccle | OV | PWNEA | top.200.illuminahiseq_rnaseq | RFS | 1096 | 0.268 | 6.8e-07 |
| CTD | dexamethasone | top.200.affymetrix_ccle | GBM | PWNEA | top.200.agilent | OS | 1940 | 0.288 | 8.3e-08 |
| CTD | dexamethasone | top.200.affymetrix_ccle | GBM | PWNEA | top.200.agilent | OS | 3881 | 0.296 | 3.4e-08 |
| CTD | dexamethasone | top.200.affymetrix_ccle | GBM | PWNEA | top.200.agilent | OS | 776 | 0.260 | 1.5e-06 |
| CGP | cisplatin | significant.filtered.  combined.maxi | LUSC | PWNEA | significant.filtered.combined.maxi | RFS | 2347 | 0.433 | 0* |
| CGP | cisplatin | significant.filtered.  combined.maxi | LUSC | PWNEA | significant.filtered.combined.maxi | RFS | 4694 | 0.433 | 0* |
| CGP | cisplatin | significant.filtered.  combined.maxi | LUSC | PWNEA | significant.filtered.combined.maxi | RFS | 938 | 0.514 | 0* |
| CTD | dexamethasone | significant.csm | GBM | PWNEA | significant.snp6 | RFS | 1940 | 0.301 | 1.9e-08 |
| CTD | dexamethasone | significant.csm | GBM | PWNEA | significant.snp6 | RFS | 3881 | 0.300 | 2.2e-08 |
| CTD | dexamethasone | significant.csm | GBM | PWNEA | significant.snp6 | RFS | 776 | 0.315 | 3.7e-09 |
| CCLE | paclitaxel | significant.  affymetrix_ccle | BRCA | PWNEA | significant.illuminahiseq_rnaseqv2 | OS | 1425 | 0.285 | 1.2e-07 |
| CCLE | topotecan | significant.  affymetrix_ccle | GBM | PWNEA | significant.agilent | RFS | 1940 | 0.363 | 6.5e-12 |
| CCLE | topotecan | significant.  affymetrix_ccle | GBM | PWNEA | significant.agilent | RFS | 3881 | 0.350 | 4.2e-11 |
| CCLE | topotecan | significant.  affymetrix_ccle | GBM | PWNEA | significant.agilent | RFS | 776 | 0.419 | 6.7e-16 |
| CCLE | paclitaxel | significant.  affymetrix_ccle | OV | PWNEA | significant.agilent | OS | 2740 | 0.345 | 7.7e-11 |
| CCLE | paclitaxel | significant.  affymetrix_ccle | OV | PWNEA | significant.agilent | OS | 5481 | 0.339 | 1.7e-10 |
| CCLE | topotecan | significant.  affymetrix_ccle | OV | PWNEA | significant.illuminahiseq_rnaseqv2 | OS | 5481 | 0.295 | 3.8e-08 |
| CCLE | topotecan | top.400.csm | GBM | GNEA | top.400.snp6 | OS | 1940 | 0.298 | 0* |
| CCLE | topotecan | top.400.csm | GBM | GNEA | top.400.snp6 | OS | 3881 | 0.313 | 0* |
| CTD | tamoxifen | top.400.  affymetrix_cgp | OV | GNEA | top.400.illuminahiseq_rnaseqv2 | OS | 2740 | 0.308 | 0* |
| CTD | tamoxifen | top.400.affymetrix_cgp | OV | GNEA | top.400.illuminahiseq_rnaseqv2 | OS | 5481 | 0.260 | 0* |
| CCLE | topotecan | top.400.affymetrix_ccle | GBM | GNEA | top.400.agilent | RFS | 1940 | 0.326 | 0* |
| CCLE | topotecan | top.400.affymetrix_ccle | GBM | GNEA | top.400.agilent | RFS | 3881 | 0.321 | 0* |
| CCLE | topotecan | top.400.affymetrix_ccle | GBM | GNEA | top.400.agilent | RFS | 776 | 0.34 | 0* |
| CCLE | topotecan | top.200.affymetrix_ccle | GBM | GNEA | top.200.agilent | OS | 776 | 0.393 | 0* |
| CCLE | topotecan | top.200.affymetrix_ccle | GBM | GNEA | top.200.agilent | RFS | 776 | 0.322 | 0* |
| CCLE | paclitaxel | top.200.affymetrix_ccle | OV | GNEA | top.200.agilent | RFS | 2740 | 0.253 | 0* |
| CCLE | paclitaxel | top.200.affymetrix_ccle | OV | GNEA | top.200.agilent | RFS | 5481 | 0.252 | 0* |
| CCLE | topotecan | top.200.affymetrix_ccle | OV | GNEA | top.200.illuminahiseq_rnaseq | OS | 1096 | 0.280 | 0* |
| CTD | gemcitabine | top.200.affymetrix_ccle | BRCA | GNEA | top.200.illuminahiseq_rnaseq | OS | 3563 | 0.274 | 0* |
| CTD | gemcitabine | top.200.affymetrix_ccle | BRCA | GNEA | top.200.illuminahiseq_rnaseq | OS | 7126 | 0.274 | 0* |
| CTD | gemcitabine | top.200.affymetrix_ccle | OV | GNEA | top.200.illuminahiseq_rnaseq | RFS | 1096 | 0.279 | 0* |
| CTD | gemcitabine | top.200.affymetrix_ccle | OV | GNEA | top.200.illuminahiseq_rnaseq | RFS | 2740 | 0.309 | 0* |
| CCLE | erlotinib | significant.filtered.  affymetrix_ccle.mini | GBM | GNEA | significant.filtered.agilent.mini | OS | 1655 | 0.317 | 0* |
| CCLE | erlotinib | significant.filtered.  affymetrix_ccle.mini | GBM | GNEA | significant.filtered.agilent.mini | OS | 3310 | 0.257 | 0* |
| CGP | docetaxel | significant.filtered.  affymetrix_ccle.mini | BRCA | GNEA | significant.filtered.illuminahiseq_rnaseqv2.mini | RFS | 1343 | 0.263 | 0* |
| CGP | docetaxel | significant.filtered.  affymetrix_ccle.maxi | BRCA | GNEA | significant.filtered.illuminahiseq_rnaseqv2.maxi | OS | 1425 | 0.271 | 0* |
| CGP | docetaxel | significant.filtered.  affymetrix_ccle.maxi | BRCA | GNEA | significant.filtered.illuminahiseq_rnaseqv2.maxi | OS | 3563 | 0.264 | 0* |
| CGP | docetaxel | significant.filtered.  affymetrix_ccle.maxi | BRCA | GNEA | significant.filtered.illuminahiseq_rnaseqv2.maxi | OS | 7126 | 0.264 | 0* |
| CGP | erlotinib | significant.filtered.  affymetrix_ccle.maxi | GBM | GNEA | significant.filtered.agilent.maxi | OS | 1940 | 0.278 | 0* |
| CGP | erlotinib | significant.filtered.  affymetrix_ccle.maxi | GBM | GNEA | significant.filtered.agilent.maxi | OS | 3881 | 0.311 | 0* |
| CGP | erlotinib | significant.filtered.  affymetrix_ccle.maxi | GBM | GNEA | significant.filtered.agilent.maxi | OS | 776 | 0.277 | 0* |
| CTD | tamoxifen | significant.  affymetrix_ccle | GBM | GNEA | significant.affymetrix | RFS | 1940 | 0.331 | 0* |
| CTD | tamoxifen | significant.  affymetrix_ccle | GBM | GNEA | significant.affymetrix | RFS | 3881 | 0.329 | 0* |
| CGP | gemcitabine | significant.  affymetrix_ccle | LUAD | GNEA | significant.illuminahiseq_rnaseqv2 | OS | 1362 | 0.308 | 0* |

*p-value below 10^-24^;

**OS: overall survival; RFS: relapse-free survival.

Supplementary Table 3. Multivariate regression models validated in ACT screen.

| **Coefficient in the model** | **AGS class** | **Data platform** | **Feature** |
| --- | --- | --- | --- |
| Nutlin | | | |
| -0.094 | mutations.mgs | Mutations_CCLE | go_bp:regulation_of_rho_gtpase_activity |
| -0.091 | mutations.mgs | Mutations_CCLE | rho-rab_tunde |
| -0.047 | mutations.mgs | Mutations_CCLE | go_bp:regulation_of_rho_protein_signal_transduction |
| -0.039 | mutations.mgs | Mutations_CCLE | alzheimercore |
| -0.03 | mutations.mgs | Mutations_CCLE | go_0043120_tnf_binding |
| -0.027 | mutations.mgs | Mutations_CCLE | go_bp:rho_protein_signal_transduction |
| -0.025 | mutations.mgs | Mutations_CCLE | biocarta_cytokine_pathway |
| 0.024 | mutations.mgs | Mutations_CCLE | kegg_04111_cell_cycle_-_yeast |
| -0.023 | significant | Affymetrix_CCLE | go_bp:interleukin_2_production |
| -0.02 | mutations.mgs | Mutations_CCLE | kegg_04744_phototransduction |
| -0.02 | mutations.mgs | Mutations_CCLE | kegg_04130_snare_interactions_in_vesicular_transport |
| -0.018 | significant | Affymetrix_CCLE | kegg_05332_graft-versus-host_disease |
| -0.017 | significant | Affymetrix_CCLE | go_0007179_tgfb_pathway |
| 0.016 | significant | Affymetrix_CCLE | go_bp:negative_regulation_of_cytokine_biosynthetic_process |
| -0.011 | significant | Affymetrix_CCLE | reactome_rho_gtpase_cycle |
| -0.0098 | significant | Affymetrix_CCLE | kegg_04940_type_i_diabetes_mellitus |
| -0.0091 | significant | Affymetrix_CCLE | sa_mmp_cytokine_connection |
| 0.0026 | mutations.mgs | Mutations_CCLE | kegg_04115_p53_signaling_pathway |
| PRIMA-1^MET^/Apr-246 | | | |
| -0.031 | significant | Affymetrix_CCLE | go_mf:rho_guanyl_nucleotide_exchange_factor_activity |
| -0.022 | significant | Affymetrix_CCLE | reactome_rho_gtpase_cycle |
| 0.013 | significant | Affymetrix_CCLE | go_0005520_igf_binding |
| -0.0091 | significant | Affymetrix_CCLE | kegg_05322_systemic_lupus_erythematosus |
| -0.0084 | significant | Affymetrix_CCLE | kegg_04612_antigen_processing_and_presentation |
| -0.0068 | significant | Affymetrix_CCLE | rho_associated_by_description_aa |
| -0.0064 | significant | Affymetrix_CCLE | kegg_05211_renal_cell_carcinoma |
| -0.0046 | significant | Affymetrix_CCLE | kegg_04662_b_cell_receptor_signaling_pathway |
| RITA | | | |
| -0.077 | mutations.mgs | Mutations_CCLE | go_bp:regulation_of_rho_gtpase_activity |
| -0.076 | mutations.mgs | Mutations_CCLE | go_mf:chemokine_activity |
| -0.065 | significant | Affymetrix_CCLE | biocarta_rho_pathway |
| -0.064 | significant | Affymetrix_CCLE | kegg_04950_maturity_onset_diabetes_of_the_young |
| -0.063 | significant | Affymetrix_CCLE | kegg_04142_lysosome |
| 0.057 | mutations.mgs | Mutations_CCLE | hs_gpcrs__class_a_rhodopsin-like_wp455_38917 |
| 0.024 | significant | Affymetrix_CCLE | nfkb-il8_tunde |
| -0.02 | significant | Affymetrix_CCLE | kegg_04530_tight_junction |
| 0.013 | significant | Affymetrix_CCLE | mesothelioma_cosmic |
| -0.011 | significant | Affymetrix_CCLE | e-cadherin_signaling_events |
| -0.011 | mutations.mgs | Mutations_CCLE | alzheimercore |
| -0.0086 | significant | Affymetrix_CCLE | e-cadherin_signaling_in_the_nascent_adherens_junction |
| -0.0085 | significant | Affymetrix_CCLE | stabilization_and_expansion_of_the_e-cadherin_adherens_junction |
| -0.0041 | significant | Affymetrix_CCLE | sig_regulation_of_the_actin_cytoskeleton_by_rho_gtpases |
| -0.003 | significant | Affymetrix_CCLE | kegg_04670_leukocyte_transendothelial_migration |
| JQ1 | | | |
| 0.084 | significant | Affymetrix_CCLE | kegg_05012_parkinson's_disease |
| -0.058 | significant | Affymetrix_CCLE | breast_cosmic |
| -0.049 | significant | Affymetrix_CCLE | go_bp:regulation_of_cytokine_production |
| 0.041 | significant | Affymetrix_CCLE | kegg_04140_regulation_of_autophagy |
| -0.032 | significant | Affymetrix_CCLE | kegg_05322_systemic_lupus_erythematosus |
| 0.029 | significant | Affymetrix_CCLE | kegg_04964_proximal_tubule_bicarbonate_reclamation |
| -0.026 | significant | Affymetrix_CCLE | go_0005154_egfr_binding |
| -0.026 | significant | Affymetrix_CCLE | go_bp:positive_regulation_of_cytokine_biosynthetic_process |
| 0.025 | mutations.mgs | Mutations_CCLE | hs_gpcrs__class_a_rhodopsin-like_wp455_38917 |
| 0.023 | mutations.mgs | Mutations_CCLE | kegg_04512_ecm-receptor_interaction |

Supplementary Table 4. TCGA clinical covariates used in the survival analysis.

| **Cohort** | **Full name** | **Used covariate(s)** |
| --- | --- | --- |
| BLCA | Bladder carcinoma | AJCC pathologic tumor stage |
| BRCA | Breast carcinoma | AJCC pathologic tumor stage, ER status by IHC, PR status by IHC, HER2 status by IHC |
| COAD | Colon adenocarcinoma | AJCC pathologic tumor stage |
| GBM | Glioblastoma multiforme | Initial pathologic diagnosis year, Karnofsky score |
| LUAD | Lung adenocarcinoma | AJCC pathologic tumor stage |
| LUSC | Lung squamous cell cancer | AJCC pathologic tumor stage |
| OV | Ovarian carcinoma | Clinical stage |
| PRAD | Prostate adenocarcinoma | Gleason score |

## Supplementary figures

## Supplementary Figure 1. Distributions of p-values and respective adjusted p-values of associations between molecular features and drug sensitivity, calculated for over 203 drugs from Basu et al. (2013)

A. All genes with somatic point mutations in CCLE cell lines according to the COSMIC screen (Forbes et al., 2014).

B. Gene expression values from the CCLE Affymetrix screen (Barretina et al., 2012).

P-values in A and B reflect confidence of one-way ANOVA and Spearman rank correlations, respectively. FDR represents p-values adjusted for multiple testing by Benjamini and Hochberg (1995). The adjustment was applied to each drug profile separately, hence the step-like pattern at the histogram in B.


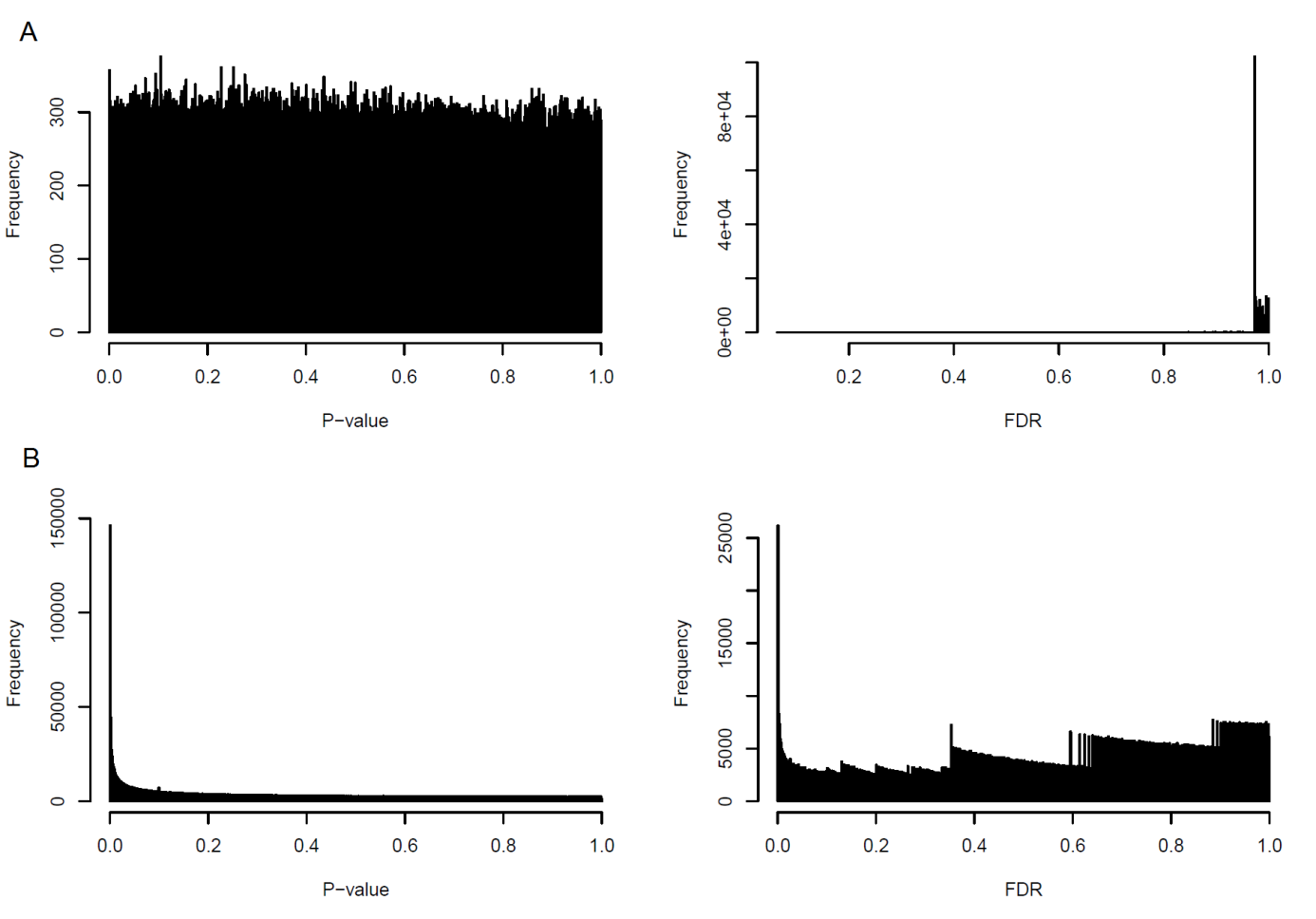


Supplementary Figure 2. Performance of different feature classes reflected by fractions of significant feature-drug associations.

See details in the legend to Fig. 2 in the main text. The data tables can be found in Supplementary_table5.FDR_rates_behind_Fig3.xlsx.

The present figure differs in presenting the full set of tested feature classes and by analysis at three different cutoffs of adjusted p-values (FDR) as FDR<0.1; FDR<0.01; and FDR<0.001.

1. Combined plot for raw data(capitalized) and NEA features (lowercase),
2. ORA features (on the same AGSs as used for NEA).

A.


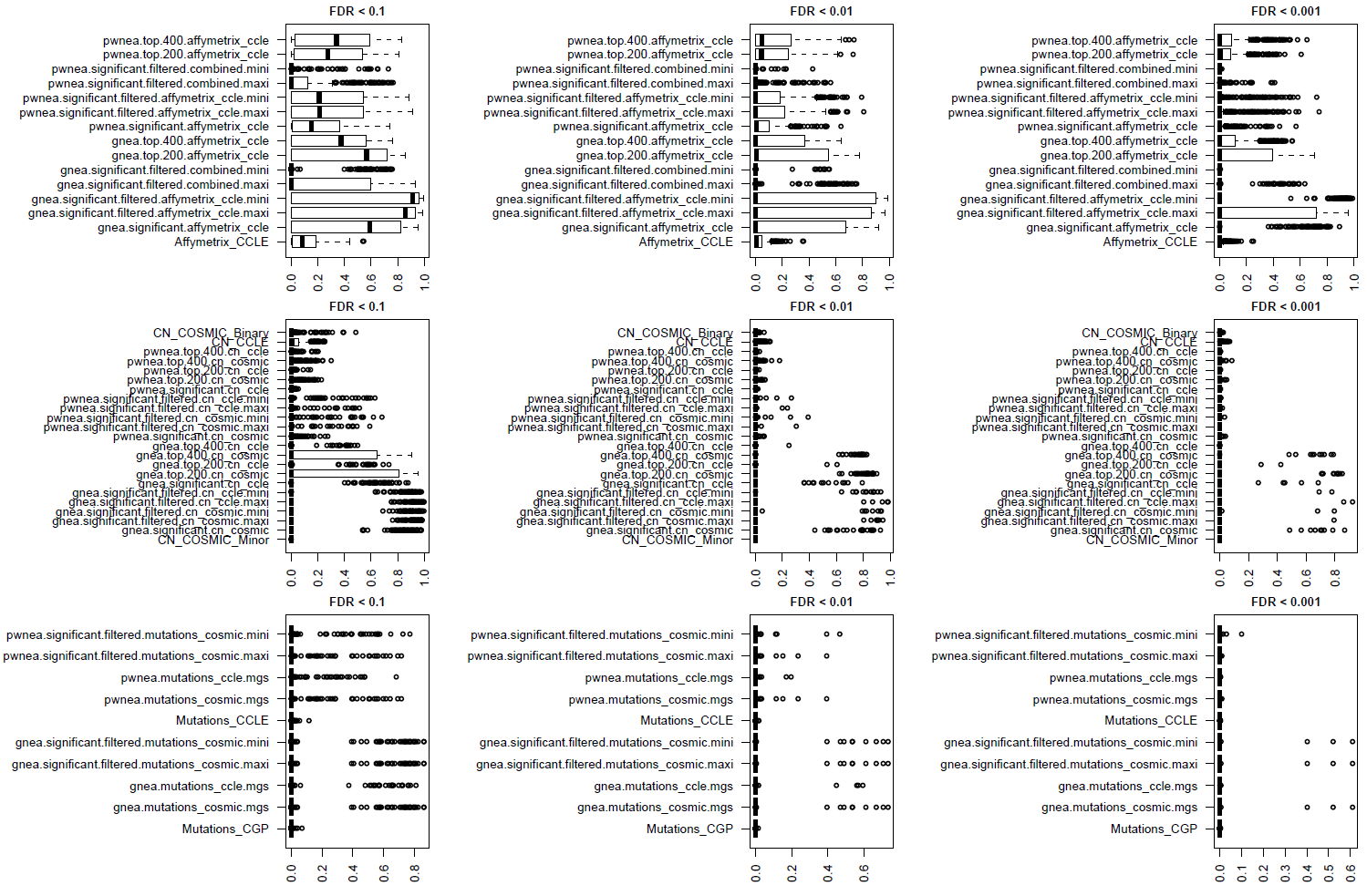


B.


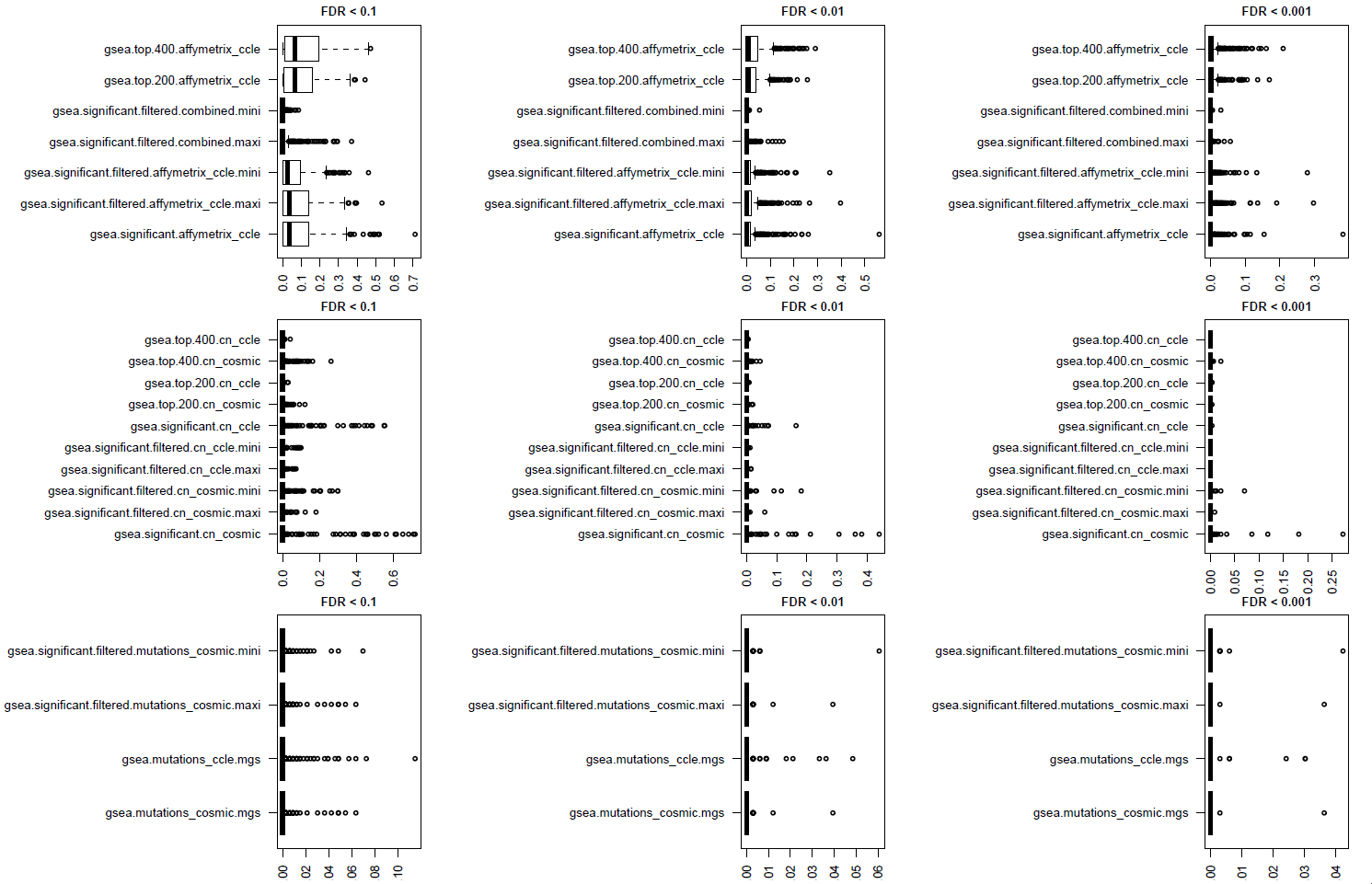


Supplementary Figure 3. Consistency of drug-association features when transferred between drug screens.

For each drug shared by any two of the three in vitro drug screens (in total 47 cases), we calculated rank correlation between respective p-values of drug-feature association (either one-way ANOVA or Spearman rank correlation) in the two screens. The figure presents fractions of cases with rank correlation above each of the 5 specified levels. NEA-based features are printed in lowercase. Original molecular features are capitalized.

See also the legend to Figure 3 in the main text.


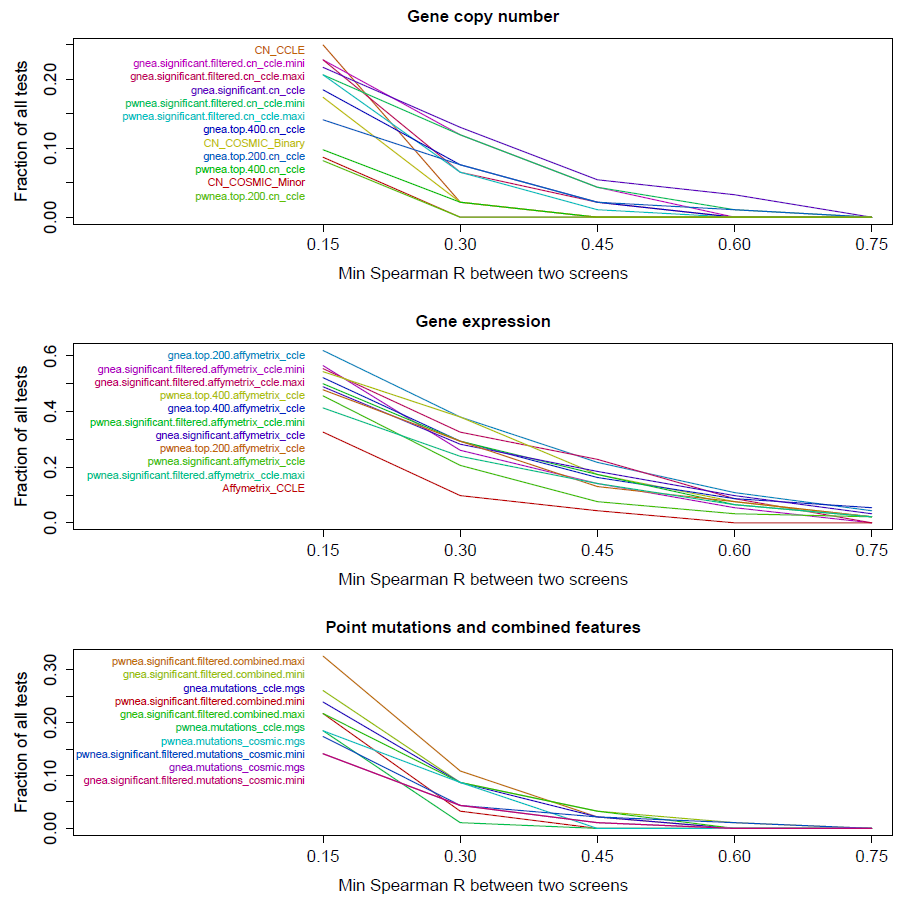

Supplement: Supplementary file 1 — Supplementary Tables And Figures [file 41598_2019_39019_MOESM1_ESM.docx]
